# An International Study of Factors Affecting Variability of Dosimetry Calculations, Part 3: Contribution from Calculating Absorbed Dose from Time-Integrated Activity

**DOI:** 10.2967/jnumed.123.267293

**Published:** 2024-08

**Authors:** Julia Brosch-Lenz, Sara Kurkowska, Eric Frey, Yuni K. Dewaraja, John Sunderland, Carlos Uribe

**Affiliations:** 1 Department of Nuclear Medicine, Rechts der Isar Medical Center, Technical University of Munich, Munich, Germany;; 2 Department of Nuclear Medicine, Pomeranian Medical University, Szczecin, Poland;; 3 Department of Integrative Oncology, BC Cancer Research Institute, Vancouver, British Columbia, Canada;; 4 Rapid, LLC, Baltimore, Maryland;; 5 Department of Radiology, Johns Hopkins University, Baltimore, Maryland;; 6 Department of Radiology, University of Michigan, Ann Arbor, Michigan;; 7 Department of Radiology, University of Iowa, Iowa City, Iowa;; 8 Molecular Imaging and Therapy, BC Cancer, Vancouver, British Columbia, Canada; and; 9 Department of Radiology, University of British Columbia, Vancouver, British Columbia, Canada

**Keywords:** radiopharmaceutical therapy, dosimetry, variability, standardization, dosimetry challenge

## Abstract

Image-based dosimetry-guided radiopharmaceutical therapy has the potential to personalize treatment by limiting toxicity to organs at risk and maximizing the therapeutic effect. The ^177^Lu dosimetry challenge of the Society of Nuclear Medicine and Molecular Imaging consisted of 5 tasks assessing the variability in the dosimetry workflow. The fifth task investigated the variability associated with the last step, dose conversion, of the dosimetry workflow on which this study is based. **Methods:** Reference variability was assessed by 2 medical physicists using different software, methods, and all possible combinations of input segmentation formats and time points as provided in the challenge. General descriptive statistics for absorbed dose values from the global submissions from participants were calculated, and variability was measured using the quartile coefficient of dispersion. **Results:** For the liver, which included lesions with high uptake, variabilities of up to 36% were found. The baseline analysis showed a variability of 29% in absorbed dose results for the liver from datasets where lesions included and excluded were grouped, indicating that variation in how lesions in normal liver were treated was a significant source of variability. For other organs and lesions, variability was within 7%, independently of software used except for the local deposition method. **Conclusion:** The choice of dosimetry method or software had a small contribution to the overall variability of dose estimates.

## Visual Abstract

**Figure:**
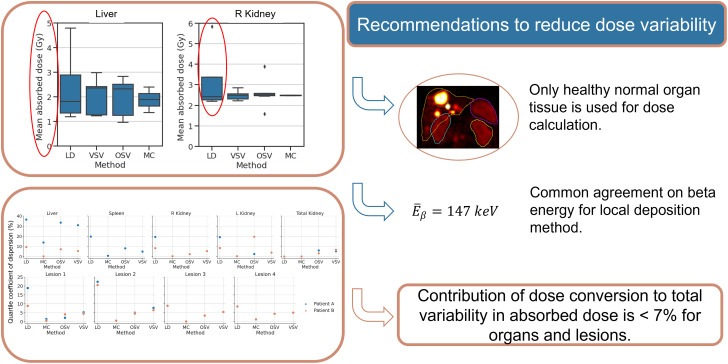

The field of radiopharmaceutical therapy is rapidly evolving, and the possible roles for dosimetry in pre- and posttherapeutic treatment planning and verification are being recognized (1*,*^2^). Patient-specific image-based dosimetry-guided radiopharmaceutical therapies have the potential to limit damage to organs at risk and maximize the therapeutic effect. The ^177^Lu dosimetry challenge of the Society of Nuclear Medicine and Molecular Imaging (SNMMI) is designed to quantitatively isolate and assess the variability contributed by each of the major steps in the determination of absorbed dose (AD) by asking participants to perform 5 tasks: region-of-interest segmentation, decay correction, curve fitting, time integration, and conversion of time-integrated activities (TIAs) to ADs. In the fifth task, both organ and lesion segmentations, as well as 3-dimensional (3D) TIA images, were provided for 2 ^177^Lu-DOTATATE SPECT/CT datasets. By separating out the variability associated with segmentation, decay correction, curve fitting, and integration methods, one can determine the remaining variability that was contributed by the actual AD calculation method.

Different dosimetry methods with varying complexity and accuracy exist (3–5). MIRD pamphlets 11 and 17 (3,4) provide general guidelines for calculating ADs at the organ or voxel level, and the European Association of Nuclear Medicine recently published a guideline for dosimetry for ^177^Lu (6). However, accurately characterizing dose–effect relationships for both organs and tumors is critical before effective clinical implementation of personalized dose prescriptions is possible. Generation of accepted dose–effect relationships will, in turn, be entirely dependent on the accuracy and comparability of AD estimates. Different steps within the dosimetry workflow will impact the final AD estimate to varying extents.

The aim of this part of the study was to understand the variability in ADs associated with the last step of the dosimetry workflow and to make recommendations related to the application of S values both at the voxel level and at the organ level.

## MATERIALS AND METHODS

### Challenge Design

The challenge design and data curation are described in our first 2 installments of this series (7*,*^8^). In brief, anonymized data from 2 patients undergoing ^177^Lu-DOTATATE therapy were made available publicly to participants through the Deep Blue Data repository of the University of Michigan (9). Sharing of patient images and data was approved by the University of Michigan Institutional Review Board, and both patients gave written informed consent. Each patient was imaged 4 times after administration over 1 wk. In task 5, participants were given access to the image data; volume-of-interest (VOI) files for kidney, liver, spleen, and lesions; and a 3D image of the TIA. Applying the VOIs to the TIA image gives organ TIAs without the variability associated with segmentation, curve fitting, and integration. Participants were asked to estimate the AD to both organs and tumors from the organ and tumor TIAs, respectively. Participants submitted their results on preformatted spreadsheets that requested details regarding their methods and intermediate results for the various steps across the different tasks. All submitted data spreadsheets for task 5 were concatenated into a single data frame collected using the Python data analysis library (Pandas, version 1.3.5) with columns corresponding to the specific variables.

### Analysis of Submissions

Submissions were grouped and categorized on the basis of user-reported values and a written description of their methodology, when provided. The categories analyzed included dosimetry method, software used, and voxel S value source. Commercial software (CS) names were pseudonymized. Further data curation was performed following the findings of our previous publication (8).

### Assessment of Variability: Baseline Data

The variability analysis was performed stepwise to establish a baseline variability in a controlled environment, to investigate the impact of the use of Radiotherapy Structure Sets (RTstructs) versus masks for the VOIs (the 2 formats used to provide segmentation to participants) and then to assess the variability in ADs from the participants.

Two medical physicists (MPs) independently performed dosimetry using the data provided in task 5 using several dosimetry software and methods that were available to both. Organ-level dosimetry using organ S values (OSVs) was tested with OLINDA (version 2.2.3) (11), IDAC-Dose (version 2.1) (11), and MIRDcalc (12). Tumors were treated as isolated spheres with unit density (OLINDA) or choice of density (IDAC-Dose and MIRDcalc). For the kidneys, the TIA was input to the software for the left, right, and total kidneys separately with their corresponding masses for the mass correction of the S values. S values are derived using human anthropomorphic phantoms and can be adjusted by multiplying by the phantom organ mass and dividing by the actual organ mass of an individual patient. 3D dosimetry using voxel S values (VSVs) was tested using ^177^Lu kernels for soft tissue that were simulated using 10^8^ primaries in GATE (version 9.2, based on GEANT4 11.0) followed by CT-based density weighting per voxel (13). Density weighting is an attempt to correct for tissue heterogeneities and uses a voxelwise density image derived from the patient’s CT image that is multiplied by the 3D VSV dose image. Lastly, individual patient Monte Carlo (MC) simulations using GATE were performed (14*,*^15^). ADs were compared per organ and per lesion between the different investigated dosimetry approaches by each of the 2 MPs and between the submitted data of task 5.

**NOTEWORTHY**

- What is the contribution of the dose conversion step to the total variability in AD estimates in radiopharmaceutical therapy when different methods and software are used at different centers?
- The contribution of the dose conversion step to the total variability in AD was found to be smaller than 7% for organs and lesions. Only healthy normal-organ tissue should be counted in the volume for dose calculation when lesions are present within an organ.
- The analysis of task 5 of the SNMMI ^177^Lu dosimetry challenge data has demonstrated that neither dosimetry method nor software had a larger impact on variability than other factors. The information on sources of variability is important for reducing variability in dose estimates and thus improving the utility of routine dosimetry and dosimetry-based personalization of radiopharmaceutical therapies.

The segmentation of organs for the SNMMI ^177^Lu dosimetry challenge was performed for each imaging time point separately, and small differences could be found across the 4 SPECT/CT studies. The challenge inadvertently introduced variability through the provision of VOIs in both RTstruct and binary-mask formats (8), leading to slightly different VOIs because the 2 formats are interpreted differently by various software. Specifically, RTstruct files contain coordinates of the vertices of polygonal VOIs and thus allow definition at the subvoxel level in a way that depends on the implementation and settings of the software used to apply VOIs to the images. On the other hand, binary masks are digitized representations that unambiguously define the voxels included in the VOI. Both were provided since not all software supports both methods. To investigate the contribution of the different VOI formats to the variability of the calculated AD, one MP applied all possible combinations of RTstructs and masks at all time points to the provided TIA image of patient A and calculated the resulting ADs using IDAC-Dose (version 2.1) to understand and report the impact of VOI formats on observed AD variabilities.

### Assessment of Variability: Participant Data

For the data from participants, general descriptive statistics (mean, SD, quartiles) of AD were calculated for liver, spleen, kidneys, 2 prespecified soft-tissue tumors in patient A, and 4 prespecified tumors in patient B. The quartile coefficient of dispersion (QCD) was calculated as the ratio of the difference and sum of the 75th and 25th quartiles. The QCD was used to assess the variability of AD results between methods and software (8). The QCD was chosen in this analysis since it is less sensitive to data outlies than is the coefficient of variation. The QCD multiplied by 1.4826 is equal to the coefficient of variation for a normal distribution. A statistical analysis compared the median of the 2 distributions of ADs from voxel- and organ-level dosimetry, and separately between commercial and non-CS, using the Mann–Whitney *U* test.

## RESULTS

### Assessment of Variability: Baseline Data

**FIGURE 1.**
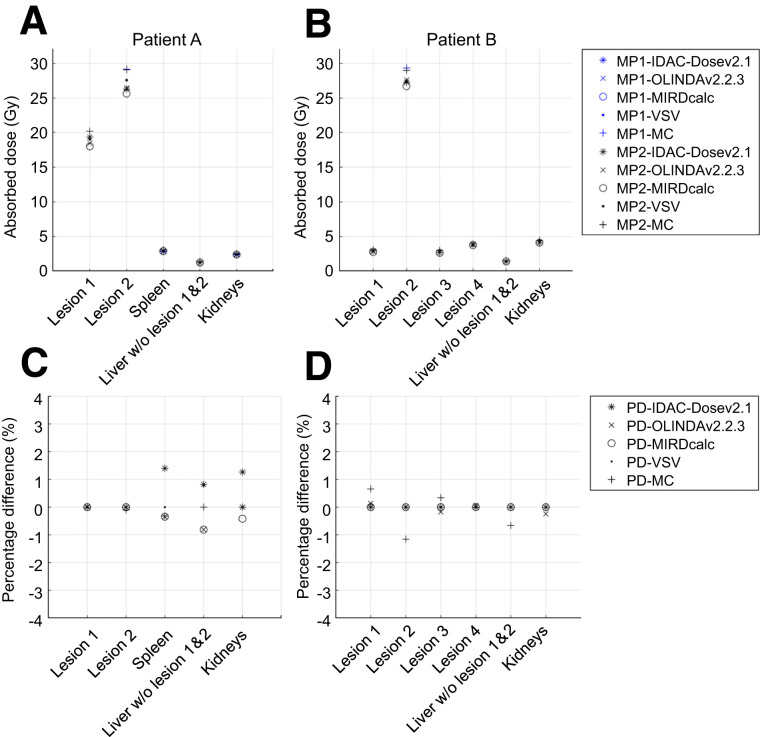
(A and B) Dosimetry results for patients A (A) and B (B), when performed by 2 MPs using same dosimetry methods. (C and D) Percentage difference in ADs between 2 MPs for patients A (C) and B (D). PD = percentage difference.

The first part of our analysis addressed the question of how different dosimetry methods affect variability when the same person or similarly trained persons (2 MPs) perform dosimetry on the same input data. Figure 1 shows the results for the ADs calculated using different OSVs, voxelized S value kernels, and full MC simulation when the procedure was performed by the 2 MPs. Only small variations were found between the 2 MPS and among the 5 investigated dosimetry approaches and software. The percentage difference in ADs reported by the 2 MPs were between −1% and +2% for the organs and between −2% and +1% for the lesions (Figs. 1C and 1D). These variations are attributed to the way each MP rounded values.

The impact on variability from using either the provided VOIs as RTstructs or masks on the TIA image of task 5 was assessed for patient A by one MP. Furthermore, the impact on the AD estimates was investigated when no mass scaling was used. Since the liver of patient A contained lesions, we investigated the effect in the ADs when lesions were included or excluded as part of the liver when performing the calculation.

**FIGURE 2.**
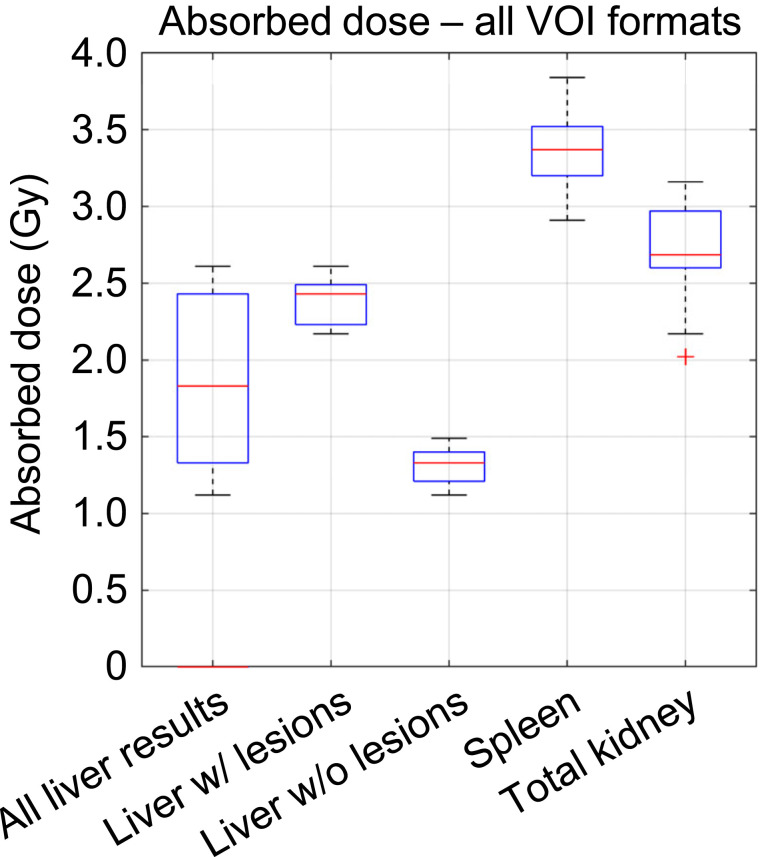
Range of ADs per organ for all possible combinations of VOI formats and scan time point used for patient A. “All Liver Results” illustrates range of liver AD results when results from both scenarios—with and without lesions included inside liver—were combined.

We noted that there were 18 combinations of how ADs could be calculated with the provided data. Use of the 2 VOI formats (i.e., mask and RTstruct) for each of the 4 time points, with and without mass scaling, provided the first 16 cases. The last two used the organ mass averaged through the 4 time points for each of the 2 VOI formats. Supplemental Figure 1 shows the differences in VOI volume over the scan time point and VOI format (supplemental materials are available at http://jnm.snmjournals.org). The AD results per organ for all 18 combinations are given in Supplemental Figure 2. The largest ranges in ADs were found for the liver, when all results of scenarios that removed lesions from the healthy liver tissue AD calculation and those that included them were combined. The range of ADs for all organs, including the different scenarios for how the liver was handled, are shown in Figure 2. Differences of 0.9–1.5 Gy were observed for the liver for all scenarios combined, as well as for spleen and total kidney.

**FIGURE 3.**
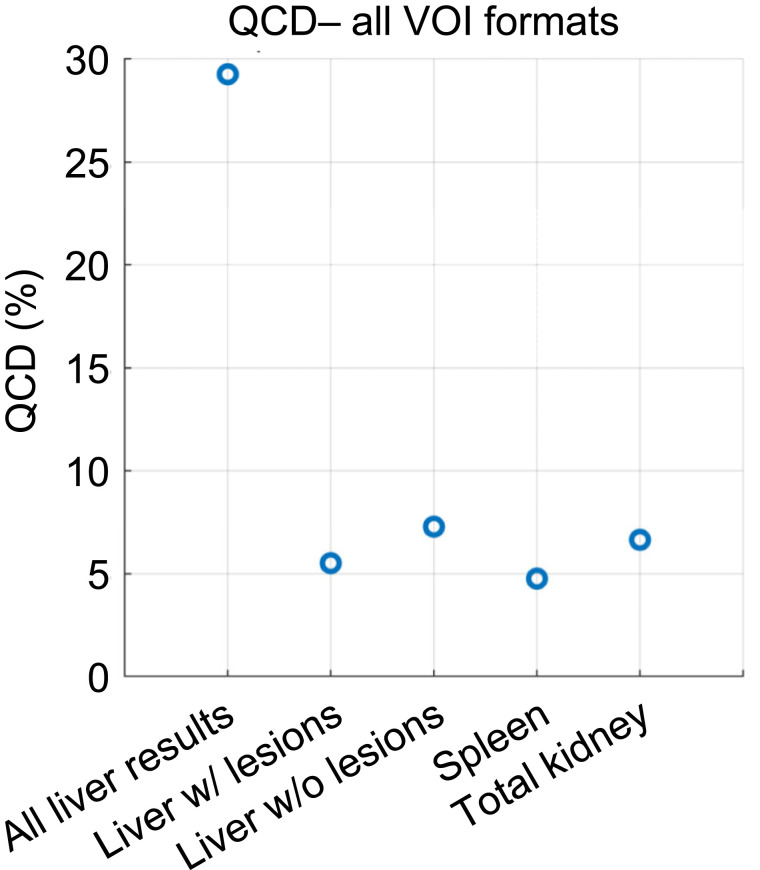
QCD for all possible 18 combinations of VOI formats and time points, with or without mass scaling, or using average mass for mass scaling, used to yield ADs in Figure 2. Furthermore, combination of all liver results—with and without lesions included—are given in “All Liver Results.”

Figure 3 provides the QCDs for all 18 results of Figure 2, when performed by one MP. The largest QCD, 29%, was found when all liver results were combined—independently of whether the liver lesions were removed. For all other organs, the QCDs ranged between 5% and 7%, indicating that VOI format (i.e., mask vs. RTstruct), selection of the time point to use for VOI definition, and application or omission of mass scaling of S values result in AD differences of up to 7%.

### Assessment of Variability: Participant Data

After the data curation, there were a total of 25 submissions per patient from 23 institutions. Table 1 provides an overview of the different dosimetry methods and software used by the participants as indicated in their submissions. There were 15 non-CS and 10 CS solutions. Overall, 13 submissions used organ-level approaches (10 OSV methods plus 3 organ-level local deposition methods), whereas 12 submissions performed voxel-level dosimetry (9 VSV methods, plus 2 MC methods and 1 voxel-level local deposition method).

**TABLE 1.** Number of Submissions per Patient for Each Dosimetry Method and Software

Parameter	Submissions (*n*)
Dosimetry method	
OSV	10
VSV	9
Local deposition (organ and voxel level)	4
MC simulation	2
Dosimetry software	
Noncommercial	15
CS 1	4
CS 2	3
CS 3	2
CS 4	1

**FIGURE 4.**
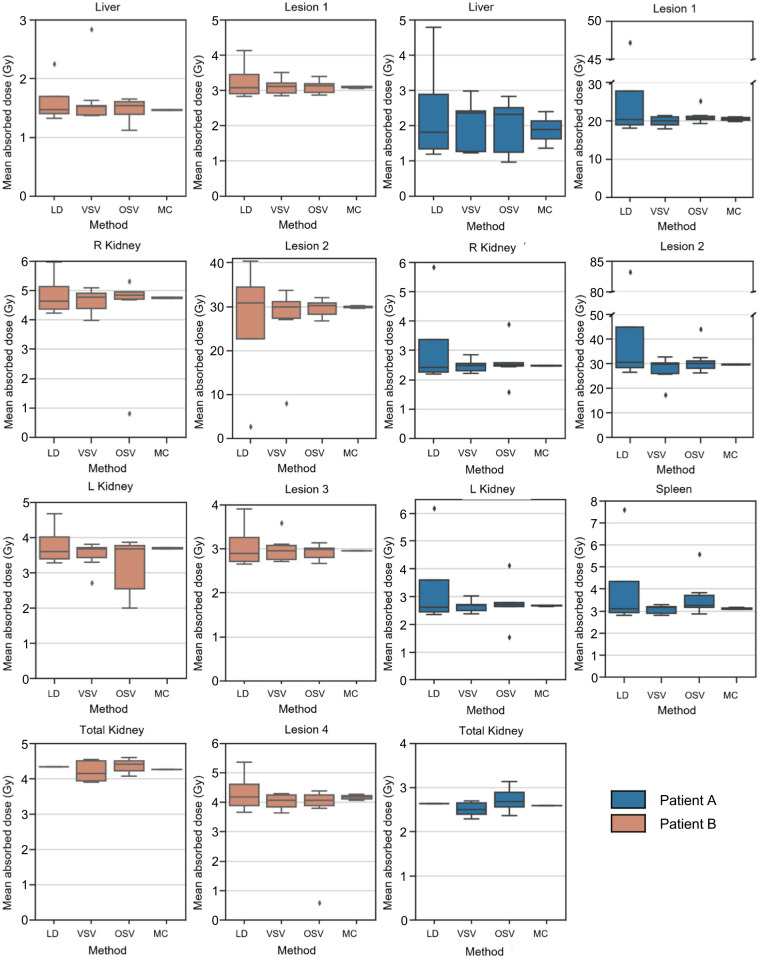
Mean AD per region for different methods that were used for calculation. Number of submissions that included each method is provided in Table 1. LD = local deposition; MC = MC dosimetry simulations; OSV = organ S value; VSV = voxel S value–based dosimetry.

**FIGURE 5.**
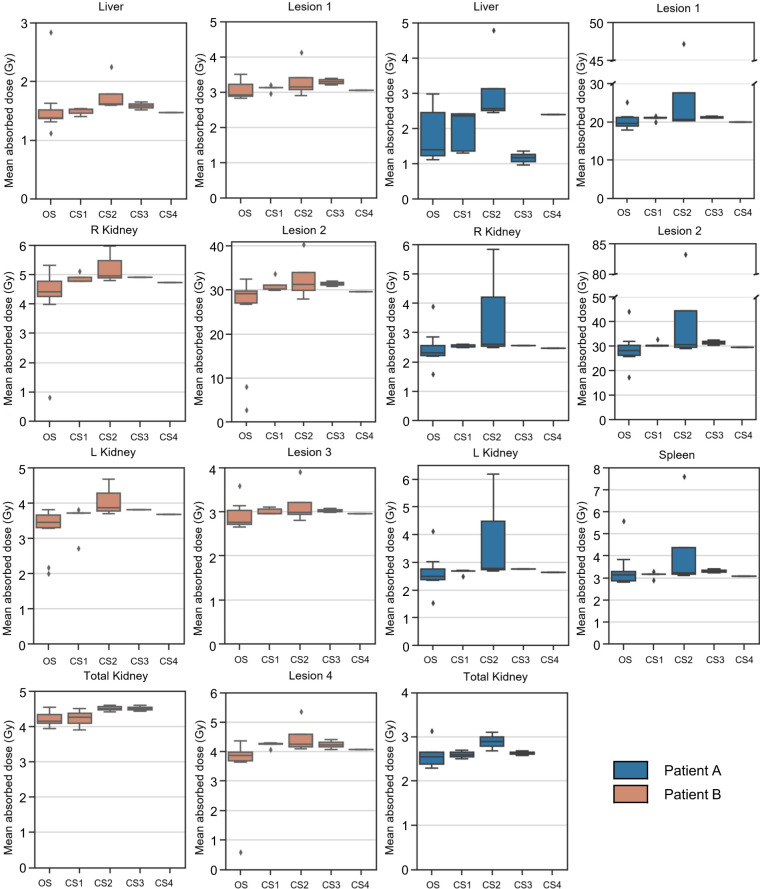
Mean AD per region using different available software solutions for all regions of patients A and B. OS = open-source/freely available and well-validated dosimetry non-CS, including in-house solutions.

The box plots in Figures 4 and 5 represent the ADs reported for the different dosimetry methods and software, respectively, for each of the VOIs provided to participants. Overall, the local deposition method showed the largest variability in ADs (Fig. 4), as illustrated by the biggest dispersion of the box plots. The MC method, with only 2 submissions, showed the smallest range in the box plot. The comparable larger range in ADs for healthy organs using the OSV method may be related to whether mass scaling of the OSV was applied. The different commercial dosimetry software yielded comparable ADs as illustrated by the comparable medians and dispersion of the ADs in the box plot in Figure 5, respectively, independently of the method, except for CS 2, which provides organ-level (using OSVs) and voxel-level (using local deposition method) dosimetry, leading to the larger range of ADs. The non-CS showed a higher variability as indicated by the second largest range of the box plot in Figure 5. This can be attributed to the use of multiple different dosimetry methods when in-house solutions are used. In the baseline analysis, we included well-known freely available and validated dosimetry software, including IDAC-Dose and MIRDcalc.

The results of the statistical analysis between voxel- and organ-level dosimetry are given in Table 2. For this test, all submissions performing 3D dosimetry (i.e., voxel level; *n* = 12) were grouped, and all organ-level submissions (*n* = 13) were grouped and compared against each other. Statistically significant differences (*P* ≤ 0.05) were found for spleen (patient A) and right and left kidneys (both patients). No statistically significant differences were found for lesions, liver, and total kidney.

**TABLE 2.** *P* Value Results of Mann–Whitney *U* Test Between Voxel- and Organ-Level Dosimetry

Patient	Liver	Spleen	R kidney	L kidney	Total kidney	Lesion 1	Lesion 2	Lesion 3	Lesion 4
A	0.29	<0.01*	0.04*	0.01*	0.30	0.07	0.08		
B	0.30		0.01*	0.05*	0.28	0.15	0.18	0.38	0.18

* Statistically significant difference (*P* ≤ 0.05).

The results of the statistical analysis between CS and non-CS (in-house solutions, well validated software, and open-source software) are given in Table 3. For this test, all submissions using CS (*n* = 10) were grouped, and all open-source software submissions (*n* = 15) were grouped and compared against each other. Statistically significant differences (*P* ≤ 0.05) were found for most lesions of both patients and the normal organs of patient B except for the total kidney. No statistically significant differences were found for the normal organs of patient A, total kidney, and lesions 1 and 3 of patient B.

**TABLE 3.** *P* value Results of Mann–Whitney *U* Test Between CS and Non-CS

Patient	Liver	Spleen	R kidney	L kidney	Total kidney	Lesion 1	Lesion 2	Lesion 3	Lesion 4
A	0.14	0.18	0.08	0.12	0.20	0.03*	0.01*		
B	0.01*		0.03*	0.02*	0.19	0.07	0.05*	0.06	<0.01*

* Statistically significant difference (*P* ≤ 0.05).

**FIGURE 6.**
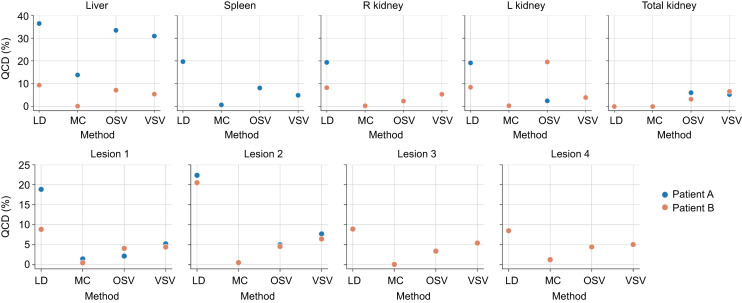
QCD per organ for different dosimetry methods. LD = local deposition; MC = MC dosimetry simulations.

**FIGURE 7.**
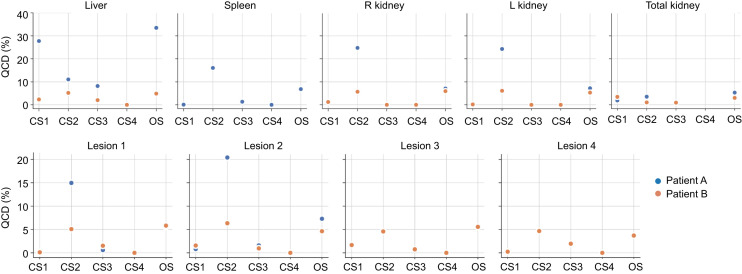
QCD per organ for different dosimetry software. OS = open-source/freely available dosimetry software, including in-house solutions and well-validated software.

Figures 6 and 7 show the QCD results between the dosimetry methods and software. The largest QCDs, up to 36%, were found for the liver for most dosimetry methods and software; the smallest QCDs, only 1% (except for liver, with 14%), were found for the MC dosimetry method, with only 2 submissions, followed by VSVs for healthy organs with QCDs of up to 7% (liver ≤ 31%) and the S value approach for lesions with QCDs below 5%. Among dosimetry software, CS 2 and the non-CS approaches showed the largest QCDs, 25% (liver, 11%) and 7% (liver, 34%), respectively, although different dosimetry methods were used.

## DISCUSSION

This analysis focused on task 5 of the SNMMI ^177^Lu dosimetry challenge based on 2 ^177^Lu-DOTATATE patient datasets with the aim of acquiring a better understanding of the variability and the sources of this variability in the last step of the dosimetry workflow, conversion of TIAs to ADs. We evaluated the use of OSVs, VSVs, local deposition method, and MC simulations.

We assessed the baseline variability in AD when performed by 2 independent MPs using the same set of dosimetry methods and software. When the same corrections were applied such as mass scaling and density weighting, the differences in ADs between methods and software were low, ranging from −1% to +2% for healthy organs and from −2% to +1% for the lesions (Fig. 1). This demonstrates that consistent ADs between different experts can be obtained independently of software and method when mass scaling and similar VOIs (6*,*^13^) are used. Note that this analysis was limited to soft-tissue structures, for which the impact of tissue heterogeneity is likely to be small. We did not evaluate tissue such as lungs, bone, and (particularly) spongiosa regions relevant for bone marrow dosimetry.

A separate analysis investigated the effect on AD variability of different formats for provision of VOIs—that is, RTstruct and binary masks, selection of VOI time point, and use or nonuse of mass scaling on OSVs. The analysis was performed only for healthy organs of patient A. The lesions were not assessed, since the VOIs were defined by a radiologist on the CT of a single time point and then copied to subsequent time points, whereas for healthy organs, CT-based segmentation was performed at each scan time point.

Overall, the up to 7% variability as assessed by QCD for kidneys and spleen was due to the 2 VOI formats, the different VOIs per scan time point, and the fact that S value mass scaling was not used in all cases (Fig. 2). The largest dispersion in ADs was found for the liver, where different scenarios with and without inclusion of the liver lesions in the liver TIA were assessed (Fig. 2). For the liver, a QCD of 29% was found when all AD data were combined—that is, AD data derived from including and excluding lesions in the overall liver VOI. The findings for the liver from the baseline variability analysis were also observed in data from participants, where QCDs of up to 36% were found for most dosimetry methods and software, which can likely be attributed to averaging of lesion dose with healthy liver dose (Figs. 6 and 7). The provided VOIs in tasks 4 and 5 of the challenge did not automatically remove the lesion uptake from the liver. A region was given for the whole liver and for individual lesions, and the participants needed to decide whether and how to remove the lesions in the liver. These findings indicate a strong need for a standardization on treatment of lesion uptake in normal tissues, especially for small organs, where the effect is assumed to be larger.

There was some variability resulting from the fact that some participants used RTstruct VOIs whereas others used binary masks. The binary masks were generated from the RTstruct VOIs and had no ambiguity about the SPECT voxels to be included. However, the application of RTstructs, which are based on polygonal contours, to SPECT images to sum voxel values has some ambiguity in terms of which fraction of voxels at the edges of the VOI should be included. How this is done is an area that would seem to be a good target for standardization to remove this source of variability.

Furthermore, VOIs for the same organ can be different for the different imaging time points depending on how they are defined. It would be beneficial for the field if recommendations could be provided as to whether individual VOIs should be obtained for each separate time point or whether only one segmentation should be performed and transferred to other time points (assuming good registration).

Finally, we observed that some of the variability in ADs is caused by use or nonuse of mass scaling for OSVs. Mass scaling is essential in obtaining accurate dose values for OSVs by adapting standard-phantom S values to patient-specific organ masses and should always be performed. If VOIs are generated for each time point, we recommend using a mass of the organ that is the average of the different VOIs.

For the analysis of the submissions from participants, the local deposition method showed the largest dispersion in ADs (QCD ≤ 36%) (Fig. 6). It is likely that this finding is due to different values of the mean energies of the ^177^Lu β-emissions used by participants (e.g., 134 vs. 147 keV (6) or not specified). This clearly requires standardization and should include the energy of the Auger electrons that are also emitted by ^177^Lu. Smaller variability in ADs was found for the VSV method (Fig. 4). The remaining dispersion in values could potentially be attributed to the use of different sources of VSVs, the application of density weighting, or—to a lesser extent given that here we are dealing with soft tissue—whether different VSVs were used for different tissue types. Two submissions used MC simulations with different MC codes, and the results were comparable.

The common approach of OSVs showed comparable results to the VSV approach (Fig. 4). It is not surprising that the OSV approach yielded the second smallest variability for the lesions, since this approach treats lesions as isolated spheres (10–12) and spheric S values seem to be well standardized. However, differences between ADs of lesions obtained with OSVs and voxel-level approaches exist because the former accounts for cross dose as well as tumor heterogeneity.

The use of commercial dosimetry software yielded comparable results (Fig. 5), with QCDs smaller than 3% except for the liver (Fig. 7). However, QCDs of CS 2 were larger, up to 25%, suggesting that the end user choose between organ- and voxel-level dosimetry in this software package, limiting the interpretation of the results for this CS. Because of the lack of further details provided by the participants, we were unable to distinguish between organ- and voxel-level dosimetry using this software. The use of non-CS (in-house solutions or freely available) showed a larger range in ADs (Fig. 5). However, this group was quite diverse in terms of the codes, methods, and level of validation. Despite this approach, these methods still provided QCDs smaller than 7% (Fig. 6, except for liver), indicating that the choice of dosimetry method or software might not be the largest source of variability in the dosimetry workflow. Many of the freely available software are extensively validated against the literature (11*,*^12^).

Comparing voxel-level versus organ-level dosimetry approaches, we found statistically significant differences among the ADs for healthy organs, whereas the differences among lesion ADs were not statistically significant (Table 2). These findings are surprising, since one would expect differences in AD for lesions when 3D methods are chosen that are capable of accounting for cross dose, whereas organ-level approaches rely on isolated spheres as representation for lesions. In contrast, there were statistically significant differences for the spleen and kidneys, even though cross dose is included in the organ-level calculation. However, these results must be treated with caution because of the small number of submissions (*n* = 25) per patient and because not all submissions included results for left kidney, right kidney, and total kidney. For the comparison of CS and non-CS, statistically significant differences in ADs were found for most lesions of both patients and the normal organs of patient B except for the total kidney. No statistically significant differences were found for normal organs of patient A or for total kidney and lesions 1 and 3 of patient B. This indicates that the dosimetry software itself may not introduce statistically significant differences in AD for all patients and organs and lesions.

Generally, we can say that the overall variability as assessed by the QCD was below 7% for all dosimetry methods, software, and regions except for the liver in patient A (Figs. 6 and 7), CS 2, and the local deposition method. These findings are in concordance with previous work that addressed the differences in ADs using different methods and software (16–19).

The small number of submissions and the fact that only 2 patient cases were provided during the challenge are limitations of our study. Furthermore, the organs and lesions included in the challenge were limited to soft-tissue structures, where differences in dosimetry methods can have a smaller impact than in heterogeneous tissue. We are aware that not all possible relevant patient characteristics were covered by these 2 cases. The issue of providing VOIs in RTstruct and mask format across the multiple imaging time points is another source of variability that is specific to this challenge. This resulted in differences in ADs of up to ±20% for the different organs. We believe that this underlines the need for standardization of VOI formats and segmentation in general to avoid such differences in volumes and therefore masses.

This analysis has shown that participants made several choices within the dosimetry workflow that increased the variability of the AD results. We believe that standardization can reduce the variability of dose calculations. We therefore recommend adoption of the following best practices.

(1) For MIRD-style, organ-level dosimetry, mass scaling is essential to account for differences between the patient’s organ mass and the mass of the stylized phantoms from which S values are precalculated.

(2) For voxelized approaches other than MC dosimetry simulation, voxel density based on registered and resampled CT images should be used to take into account patient-specific tissue heterogeneities.

(3) We found variabilities in ADs of up to 36% for the liver depending on whether lesions were included or excluded from the VOIs. For ^177^Lu, the AD from the liver excluding lesions is likely to be a better predictor of organ toxicity than if the lesions are included. We thus recommend standardization that excludes lesions present in the organ from VOIs used to estimate AD to healthy tissues using the OSV approach. 3D dosimetry methods can account for cross irradiation of lesions to healthy tissue, and VOIs for healthy tissue should consequently exclude the lesion VOI to extract mean ADs from 3D dose images.

(4) Local energy deposition methods showed high variability because different mean energies for the ^177^Lu electron emissions were used. We recommend using a mean energy of 147.1 keV Bq^−1^ s^−1^ as specified in several guidelines (6*,*^20^).

(5) We observed significant variability when applying VOIs given as binary masks versus polygonal contours (as RTstruct). This could be related to differences in how polygonal masks defined at a higher resolution (e.g., using CT images) are applied to SPECT images with larger voxels and on how subvoxel contours are in general compared against binary masks. Standardization of the VOI format is needed for the application in dosimetry.

(6) It is imperative not just to report ADs but to also include a detailed description of the methods used such that if discrepancies between reported values are present, those differences can be understood and harmonization of results pursued.

(7) Widely used software, both CS and freely available, resulted in ADs that were comparable to each other. One explanation is that this software is better validated. There was greater variability in results calculated using less widely used software. Use of software that is well validated is thus highly recommended.

## CONCLUSION

The analysis of task 5 of the SNMMI ^177^Lu dosimetry challenge data based on ^177^Lu-DOTATATE patients has demonstrated that the contribution to variability from the dosimetry methods and software used are small and that other possible factors such as segmentation and curve fitting and integration have a higher impact. The contribution of the dose conversion step to the total variability in AD was found to be smaller than 7% for lesions and organs not containing lesions. We have made some recommendations that, if adopted widely by the community, can further reduce variability in AD results.

## DISCLOSURE

This work was partly supported by the SNMMI Value Initiative. Yuni Dewaraja acknowledges funding from grant R01CA240706 awarded by the National Cancer Institute for the patient studies and the resources made available by the University of Michigan Deep Blue Data Repository for data sharing. Eric Frey is a cofounder and part-owner of Rapid, LLC, receives royalty income from GE Healthcare, and acknowledges support from grants R44CA213782 and R01CA240779 awarded by the National Cancer Institute. Carlos Uribe acknowledges funding from Natural Science and Engineer Research Council (NSERC) discovery grant RGPIN-2021-02965. No other potential conflict of interest relevant to this article was reported.
